# A self-assembling nanomedicine of conjugated linoleic acid-paclitaxel conjugate (CLA-PTX) with higher drug loading and carrier-free characteristic

**DOI:** 10.1038/srep36614

**Published:** 2016-11-04

**Authors:** Ting Zhong, Xin Yao, Shuang Zhang, Yang Guo, Xiao-Chuan Duan, Wei Ren, Dan Huang, Yi-Fan Yin, Xuan Zhang

**Affiliations:** 1 Beijing Key Laboratory of Molecular Pharmaceutics and New Drug Delivery Systems, School of Pharmaceutical Sciences, Peking University, Beijing 100191, China; 2 Department of Pharmaceutics, School of Pharmaceutical Sciences, Peking University, Beijing 100191, China

## Abstract

The main objective of this study was to demonstrate the proof-of-principle for the hypothesis that conjugated linoleic acid-paclitaxel conjugate (CLA-PTX), a novel fatty acid modified anti-cancer drug conjugate, could self-assemble forming nanoparticles. The results indicated that a novel self-assembling nanomedicine, CLA-PTX@PEG NPs (about 105 nm), with Cremophor EL (CrEL)-free and organic solvent-free characteristics, was prepared by a simple precipitation method. Being the ratio of CLA-PTX:DSPE-PEG was only 1:0.1 (w/w), the higher drug loading CLA-PTX@PEG NPs (about 90%) possessed carrier-free characteristic. The stability results indicated that CLA-PTX@PEG NPs could be stored for at least 9 months. The safety of CLA-PTX@PEG NPs was demonstrated by the MTD results. The anti-tumor activity and cellular uptake were also confirmed in the *in vitro* experiments. The lower crystallinity, polarity and solubility of CLA-PTX compared with that of paclitaxel (PTX) might be the possible reason for CLA-PTX self-assembling forming nanoparticles, indicating a relationship between PTX modification and nanoparticles self-assembly. Overall, the data presented here confirm that this drug self-delivery strategy based on self-assembly of a CLA-PTX conjugate may offer a new way to prepare nanomedicine products for cancer therapy involving the relationship between anticancer drug modification and self-assembly into nanoparticles.

The rapid growth in nanotechnology involving the development of anticancer nanomedicines has led to great improvements in therapeutic strategies to treat cancer^1^,^2^,^3^. Now, a wide range of nanomaterials based on organic, inorganic, lipid, protein, or polymeric compounds are being used as carrier materials to prepare new anticancer nanomedicine systems^2^.

Ideally, anticancer nanomedicine products should have a high drug loading to avoid administering excessively high amounts of carrier material, involve safe and nontoxic carrier materials, and improve the therapeutic effect of the anticancer agent as well as being easy to prepare^4^. In fact, very few anticancer nanomedicine systems are able to meet the above requirements.

It has been reported that polymer-drug conjugates can self-assemble into nanomedicines with well-defined sizes and shapes using a precipitation procedure^5^,^6^,^7^. Unlike traditional carrier-based nanomedicines, polymer-drug conjugates do not require the use of additional carriers. The drug loading capacity can also be very high (up to 100% if the nanostructure is made of the conjugate itself). Preparation by precipitation is an easy procedure which involves (1) dissolving the conjugate in an organic solvent, (2) then mixing the homogeneous solution with water, and (3) after the nanomedicine is self-assembled, the organic solvent can be removed to form a dispersion of spherical nanoparticles in aqueous solution. If the conjugate is designed correctly, factors such as the conjugate concentration, solvent choice, titration or mixing rate, and agitation play an important role in determining the size and shape of the nanostructures^8^. Now, many polymer-drug conjugates have been reported to self-assemble forming nanomedicines, including micelles^9^ or nanogels^10^. However, since the molecular weight of the polymer is higher than that of the anticancer drug, the drug occupancy rate in these polymer-drug conjugates is not very high, such as polyethylene glycol-paclitaxel (PEG-PTX) conjugate (drug occupancy rate 6.0%)^11^, hydroxypropyl methacrylate-paclitaxel (HPMA-PTX) conjugate (drug occupancy rate 7.3%)^12^ and cholesteryl-hyaluronic acid-salinomycin (CHA-SAL) conjugate (drug occupancy rate 21.6%)^13^.

In order to overcome this problem, some small molecule modified anti-cancer drug conjugates with amphiphilic characteristic were reported to self-assemble into nanoparticles in water, which were driven largely by the amphiphilicity of the conjugates, such as the short oligomer chain of ethylene glycol-camptothecin (camptothecin occupancy rate of 58.5%) which could assemble into liposome-like nanocapsules with diameter of 132 nm and zeta potential of −4.0 mV^14^, the squalenoyl-doxorubicin (doxorubicin occupancy rate 57%) that was able to form “loop-train” nanoassemblies with diameter of 130 nm and zeta potential of +35.5 mV^4^, and the irinotecan-chlorambucil conjugates (irinotecan occupancy rate of 67.2%) which resulted in self-assembling into nanoparticles with size of 88.3 nm and zeta potential of +3.4 mV^15^. Even more encouragingly, some small molecule modified anti-cancer conjugates with hydrophobic characteristic were proven to be able to self-assemble into nanoparticles in water. For instance, the squalenoyl-paclitaxel (paclitaxel occupancy rate 69.1%) was reported to be able to formulate into nanoassemblies with size of 117.7 nm and zeta potential of −36.88 mV, which was supposed to depend on the squalene’s molecular flexibility and dynamically folded conformation^16^. As a further example, the vitamin E-disulfide-paclitaxel (paclitaxel occupancy rate 59.7%) was proven to self-assemble into nanoparticles with diameter of 113 nm and zeta potential of −29.2 mV, which may resulted from the insertion of a single disulfide bond that balance the competition between intermolecular forces involved in the self-assembly process of hydrophobic molecules^17^.

The conjugated linoleic acid-paclitaxel conjugate (CLA-PTX) has been synthesized by us as a fatty acid modified paclitaxel conjugate (drug occupancy rate 76.5%)^18^. The *in vitro* and *in vivo* anti-tumor activity of CLA-PTX has been confirmed in C6 glioma and B16-F10 melanoma cells^18^,^19^,^20^. CLA-PTX also exhibits a higher cellular uptake in C6 glioma and B16-F10 melanoma cells compared with paclitaxel. In addition, the anti-tumor activity of a CLA-PTX microemulsion and CLA-PTX-loaded iRGD modified liposomes was also confirmed in *in vitro* and *in vivo* experiments^19^,^21^.

Since some small molecule modified anti-cancer drug conjugates can self-assemble into nanoparticles, the main objective of this study was to demonstrate the proof-of-principle for the hypothesis that CLA-PTX can self-assemble to form nanoparticles. The role of fatty acid modification of PTX in terms of self-assembling nanoparticles was investigated. A higher drug loading CLA-PTX nanomedicine was prepared by using a simple precipitation procedure. The safety and anti-tumor activity of this nanomedicine were also evaluated.

## Materials and Methods

### Materials

**Figure 1:**
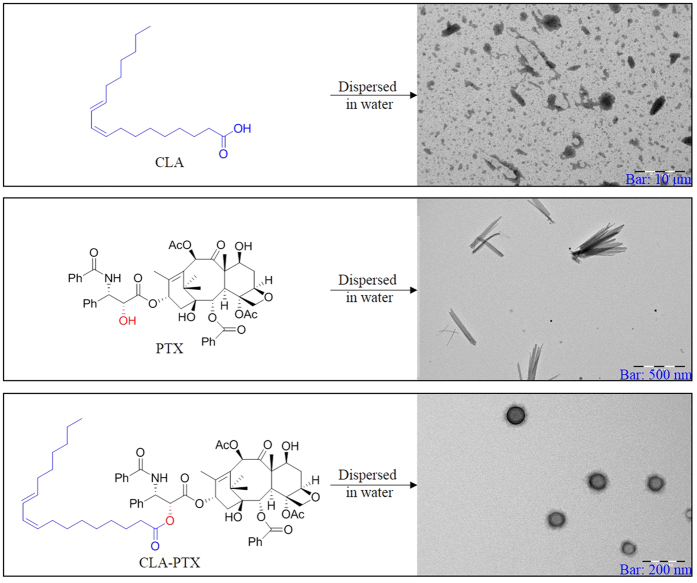
The structure of CLA, PTX and CLA-PTX and the TEM of CLA, PTX and CLA-PTX NPs.

Paclitaxel (PTX) was obtained from Ouhe Co., Ltd. (Beijing, China). Conjugated linoleic acid (CLA), N,N-dicyclohexylcarbodiimide (DCC), 4-dimethylaminopyridine (DMAP) and sulforhodamine B (SRB) were purchased from Sigma-Aldrich (St. Louis, MO, USA). CLA-PTX was synthesized from CLA and PTX according to our previous report^18^, the structure of CLA-PTX is shown in Fig. 1. 1,2-distearoyl-sn-glycero-3-phosphoethanolamine [methoxy(polyethylene glycol)_2000_] (DSPE-PEG) was obtained from NOF Co. (Tokyo, Japan). Paclitaxel injection (Taxol) was commercially available from a local Beijing hospital (Bristol Myers Squibb Co., Princeton, NJ, USA). All other chemicals were analytical grade or HPLC grade.

### Cells

Murine melanoma B16-F10 cells, human breast carcinoma MDA-MB-231cells and human brain glioma U87-MG cells were obtained from the Chinese Academy of Sciences Cell Bank (Shanghai, People’s Republic of China). These cell lines were all cultured according to the recommended instructions.

### Animals

ICR mice, 50% male and 50% female (5–6 week-old and weighing 20–22 g) were provided by the Experimental Animal Center of Peking University Health Science Center (Beijing, People’s Republic of China). All care and handling of the animals were performed with the approval of Institutional Authority for Laboratory Animal Care of Peking University.

### Solubility of CLA-PTX

The solubility of CLA-PTX was measured by the static equilibrium method with modification. In brief, an excess of CLA-PTX was added to 5 ml distilled water, then incubated in an orbital shaker at 37 °C with mild oscillation at 30 rpm for 48 h. Each sample was taken out, centrifuged at 10000 rpm for 5 min, and then passed through a polyvinylidene fluoride syringe-filter (pore size 0.22 μm). The resulting filtrates were determined by HPLC analysis.

### Preparation of CLA-PTX NPs

In brief, CLA-PTX was dissolved in dimethylsulfoxide (DMSO) to obtain a CLA-PTX DMSO solution of 1 mg/ml, then, 0.3 ml of this solution was added drop-wise to 3 ml distilled water under continuous and gentle stirring (300–500 rpm) at room temperature. Under these conditions, the self-assembly of CLA-PTX nanoparticles (CLA-PTX NPs) occurred spontaneously. Transmission electron microscopy (TEM) was used to determine the particle morphology. Similarly, CLA and PTX were handled in the same way as CLA-PTX.

### Effect of CLA-PTX concentration and added volume on the particle size of CLA-PTX NPs

The effect of the CLA-PTX concentration and added volume on the particle size of CLA-PTX NP was investigated. The concentration of CLA-PTX was 1, 2, 5, 10, 15, 20 and 30 mg/ml, respectively. According to the CLA-PTX NPs preparation, a volume of 0.3 or 1.0 ml CLA-PTX DMSO solution was added drop-wise to 3 ml distilled water under continuous and gentle stirring (300–500 rpm) at room temperature. After dialysis against distilled water for 48 h (MWCO = 3500 Da), the particle size of CLA-PTX NPs was determined by dynamic light scattering (DLS) measurements on a Nano-ZS instrument (Malvern, Worcestershire, UK).

### Effect of DSPE-PEG on CLA-PTX NPs preparation

#### Effect of added DSPE-PEG on the particle size of CLA-PTX NPs

The concentration of CLA-PTX was 1, 2, 5, 10, 15, 20 and 30 mg/ml, respectively. DSPE-PEG was added to the CLA-PTX DSMO solution at a ratio of CLA-PTX:DSPE-PEG = 1:0.1 (w/w). According to the CLA-PTX NP preparation, a volume of 0.3 or 1.0 ml CLA-PTX mixed DSPE-PEG DMSO solution was added drop-wise to 3 ml distilled water under continuous and gentle stirring (300–500 rpm) at room temperature. After dialysis against distilled water for 48 h (MWCO = 3500 Da), the particle size of CLA-PTX NPs was determined by dynamic light scattering (DLS) measurements on a Nano-ZS instrument (Malvern, Worcestershire, UK).

#### Effect of added amount of DSPE-PEG on CLA-PTX NPs

The ratio of CLA-PTX:DSPE-PEG was set at 1:0.0125, 1:0.025, 1:0.05, 1:0.1, 1:0.15, 1:0.2, 1:0.33, 1:1, 1:3, 1:5, 1:7, 1:10, 1:20, 1:40 or 1:80 (w/w). The concentration of CLA-PTX was 2 mg/ml. According to the CLA-PTX NPs prparation, a volume of 0.3 ml CLA-PTX mixed DSPE-PEG DMSO solution was added drop-wise to 3 ml distilled water under continuous and gentle stirring (300–500 rpm) at room temperature. The particle size of CLA-PTX NPs was determined by dynamic light scattering (DLS) measurements on a Nano-ZS instrument (Malvern, Worcestershire, UK).

As a control group, the CLA-PTX was replaced by PTX. Similarly, the ratio of PTX:DSPE-PEG was set at 1:0.0125, 1:0.025, 1:0.05, 1:0.1, 1:0.15, 1:0.2, 1:0.33, 1:1, 1:3, 1:5, 1:7, 1:10, 1:20, 1:40 or 1:80 (w/w). The concentration of PTX was 2 mg/ml. According to the CLA-PTX NPs preparation, a volume of 0.3 ml of PTX mixed DSPE-PEG DMSO solution was added drop-wise to 3 ml distilled water under continuous and gentle stirring (300–500 rpm) at room temperature. The particle size of the PTX particles was determined by dynamic light scattering (DLS) measurements on a Nano-ZS instrument (Malvern, Worcestershire, UK).

### Preparation of CLA-PTX@PEG NPs

CLA-PTX and DSPE-PEG (CLA-PTX:DSPE-PEG = 1:0.1, w/w) was dissolved in DMSO to obtain a concentration of CLA-PTX of 30 mg/ml. A volume of 1 ml sample of this solution was added drop-wise to 3 ml of distilled water with continuous and gentle stirring (300–500 rpm) at room temperature. Under these conditions, the self-assembly of CLA-PTX nanoparticles (CLA-PTX@PEG NPs) occurred spontaneously. Then, the resulting nanoparticle dispersion was dialyzed against distilled water for 48 h (MWCO = 3500 Da) and the obtained nanoparticle dispersion was subjected to ultra-filtration at 4000 rpm for 5 min. The concentration of CLA-PTX in the obtained CLA-PTX@PEG NPs was 6 mg/ml (if necessary, adjusted with distilled water).

### Characterization

#### Particle size and distribution

The particle size and zeta potential of the nanoparticles were determined by dynamic light scattering (DLS) measurements on a Nano-ZS instrument (Malvern, Worcestershire, UK).

#### Transmission electron microscopy (TEM)

Transmission electron microscopy (TEM) was carried out using a JEM 1400 microscope (JEOL USA, Inc., Peabody, MA) operated at 140 kV using a permeable carbon-coated copper grid.

#### X-ray diffraction

The X-ray diffraction patterns of the samples were measured using a D/MAX 2000 rotating anode X-ray diffractometer (Rigaku Co., Japan) equipped with a Cu-Kα X-ray source (λ = 1.541 nm, 40 kV /100 mA). XRD data were collected over the 2θ range from 3° to 40° at a step size of 0.02° at increments for quantitative analysis. Profile fits of the data were performed using Origin 8.5.1 software.

#### HPLC analysis

HPLC analysis was carried out using an HPLC system involving an LC-20AT liquid chromatograph (SHIMADZU, Japan) and an SPD-M20A diode array detector (SHIMADZU, Japan)^18^,^21^. Briefly, the determination was carried out on an ODS 3 C18 analytical column (5 μm, 250 × 4.6 mm; Phenomenex, Torrance, CA, USA) gradient eluting with mobile phases of acetonitrile (mobile phase A) and water (mobile phase B) in the mode (0–10 min, phase A, 60%; 10–12 min, phase A 60%→100%; 12–28 min, phase A 100%; 28–30 min, phase A 100%→60%; 30–40 min, phase A 60%) at the flow rate of 1.0 ml/min. The column temperature was set at 40 °C and the detection wavelength was set at 227 nm. The retention time of PTX and CLA-PTX was approximately 7 and 22 minutes, respectively.

### Maximum tolerated dose studies (MTD)

The maximum tolerated dose (MTD) of CLA-PTX@PEG NPs was determined using a dose escalation method in healthy ICR mice, 50% male and 50% female. Animals (n = 10, per dose group) received i.v. injections of CLA-PTX@PEG NPs (90, 180 and 270 mg/kg), Taxol (20, 25 and 30 mg/kg) or 5% glucose as a control. Animal survival and changes in body weight were observed daily over two weeks in all groups. The highest dose that did not cause toxicity (as defined by a median body weight loss of 10% of the control or abnormal behavior including hunched posture and a rough coat) was defined as the MTD.

To evaluate the toxicity occurring after treatment, the mice were sacrificed on the seventh day after administration. Blood samples were collected for hematological and biochemical analysis. The hematological indices, such as white blood cell (WBC), red blood cell (RBC), and platelet (PLT) counts, were determined in an MEK-6318 K automatic hematology analyzer (Nihon Kohden, Shinjuku-ku, Japan).

### Cellular uptake

B16-F10 cells, MDA-MB-231 cells or U87-MG cells were seeded at a density of 2.5 × 10^5^ cells/well in 6-well plates and incubated at 37 °C for 24 h to allow cell attachment. Then, cells were treated with free CLA-PTX solution or CLA-PTX@PEG NPs (10 μM) for 2, 4 or 6 h at 37 °C. The cellular uptake efficiency was determined as in our previous report^21^,^22^. In detail, after incubation, cells were washed three times with cold phosphate-buffered saline (PBS, pH 7.4), and lysed with 0.1% sodium dodecyl sulfate (SDS, 0.1 ml). Following that, 5 μl of the lysate were taken for the determination of the protein concentration using a Pierce™ BCA Protein Assay Kit (Thermo Scientific, Waltham, USA), in accordance with the manufacturer’s instructions. Then, the lysate was extracted with 0.3 ml acetonitrile, and centrifuged at 10000 rpm for 5 min. A volume of 50 μl of the resulting supernatant was used for the measure of the amount of CLA-PTX in the samples through HPLC analysis. Three wells were measured for each sample. The cellular uptake of free CLA-PTX solution or CLA-PTX@PEG NPs was calculated using the following formula:


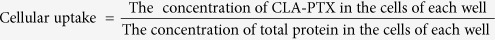


### *In vitro* cytotoxicity

The *in vitro* cytotoxicity of CLA-PTX@PEG NPs was evaluated in B16-F10 cells, MDA-MB-231 cells and U87-MG cells using the sulfonylrhodamine B (SRB) assay^18^,^21^,^23^. The half-maximal inhibitory concentration (IC_50_) was calculated according to the dose-effect curves using GraphPad Prism 6 software.

### Statistical analysis

Data are presented as the mean ± standard deviation (SD). One-way analysis of variance (ANOVA) was used to determine significance among groups, after which post-hoc tests with the Bonferroni correction were used for comparison between individual groups. Statistical significance was established at *p* < 0.05.

### Statement

In addition, all methods were performed in accordance with the relevant guidelines and regulations.

## Results and Discussion

### CLA-PTX NPs preparation

It is well known that paclitaxel (PTX) cannot self-assemble to form nanoparticles. Interestingly, the conjugated linoleic acid-paclitaxel (CLA-PTX), which we previously synthesized in which PTX was modified by conjugated linoleic acid (CLA)^18^, could self-assemble to form CLA-PTX nanoparticles (CLA-PTX NPs) with a spherical morphology when CLA-PTX DMSO solution was added to distilled water (Fig. 1). In contrast, droplets of CLA and crystal PTX were observed when CLA or PTX DMSO solutions were added to water (Fig. 1), respectively.

### Effect of CLA-PTX concentration and added volume on CLA-PTX NPs

**Figure 2:**
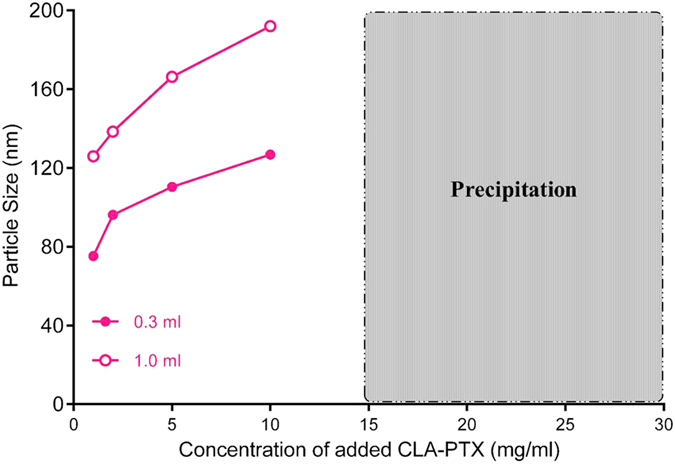
The effect of CLA-PTX concentration and added volume on the particle size of CLA-PTX NPs. A volume of 0.3 ml of a series of CLA-PTX DMSO solution added to 3 ml distilled water (pink line and pink solid circle); A volume of 1 ml of a series of CLA-PTX DMSO solution added to 3 ml distilled water (pink line and pink empty circle). The particle size of CLA-PTX NPs was determined after dialysis against distilled water for 48 h (MWCO = 3500 Da).

The effect of the CLA-PTX concentration and added volume on the particle size of CLA-PTX NPs was investigated. The results indicated that the particle size of CLA-PTX NPs increased with the concentration and added volume of CLA-PTX DMSO solution (Fig. 2). In addition, when the concentration of CLA-PTX exceeded 10 mg/ml, the CLA-PTX would precipitate out in the distilled water.

### Effect of DSPE-PEG on CLA-PTX NPs preparation

DSPE-PEG, an amphiphilic material, is an FDA-approved pharmaceutical material used for the preparation of long-circulating liposome products. It is a PEGylated phospholipid that has been widely used for the preparation of various nano-carrier formulations, such as micelles, long-circulating liposomes or lipid-protected particles, where the PEG layer often acts as a steric barrier that protects the molecular assemblies against uptake by the mononuclear phagocytic system (MPS) to produce a prolonged blood circulation time, improved stability and increased encapsulation efficiency^24^.

**Figure 3:**
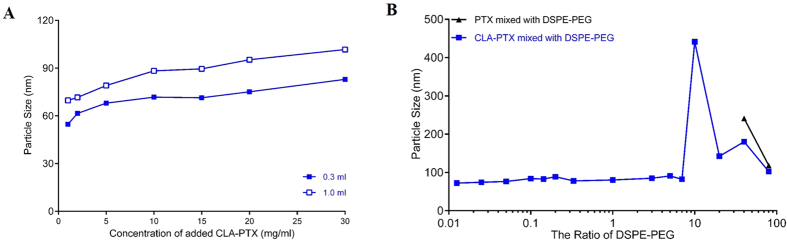
The effect of DSPE-PEG on the CLA-PTX NPs particle size. (**A**) The effect of CLA-PTX concentration and added volume as well as DSPE-PEG on the particle size of CLA-PTX NPs. A volume of 0.3 ml of a series of CLA-PTX mixed DSPE-PEG (the ratio of CLA-PTX: DSPE-PEG = 1:0.1, w/w) DMSO solution added to 3 ml distilled water (blue line and blue solid diamond); A volume of 1 ml of a series of CLA-PTX mixed DSPE-PEG (the ratio of CLA-PTX: DSPE-PEG = 1:0.1, w/w) DMSO solution added to 3 ml distilled water (blue line and blue empty diamonds). The particle size of CLA-PTX NPs was determined after dialysis against distilled water for 48 h (MWCO = 3500 Da). (**B**) The effect of the added amounts of DSPE-PEG on the CLA-PTX NPs particle size. The ratio of CLA-PTX:DSPE-PEG was set at 1:0.0125, 1:0.025, 1:0.05, 1:0.1, 1:0.15, 1:0.2, 1:0.33, 1:1, 1:3, 1:5, 1:7, 1:10, 1:20, 1:40 or 1:80 (w/w). The concentration of CLA-PTX was 2 mg/ml. According to the CLA-PTX NPs preparation, a volume of 0.3 ml CLA-PTX mixed DSPE-PEG DMSO solution was added drop-wise to 3 ml distilled water under continuous and gentle stirring (300–500 rpm) at room temperature. As a control group, the CLA-PTX was replaced by PTX. Similarly, the ratio of PTX:DSPE-PEG was set at 1:0.0125, 1:0.025, 1:0.05, 1:0.1, 1:0.15, 1:0.2, 1:0.33, 1:1, 1:3, 1:5, 1:7, 1:10, 1:20, 1:40 or 1:80 (w/w). The concentration of PTX was 2 mg/ml. According to the CLA-PTX NPs preparation, a volume of 0.3 ml of PTX mixed DSPE-PEG DMSO solution was added drop-wise to 3 ml distilled water under continuous and gentle stirring (300–500 rpm) at room temperature.

In the present research, we observed that the particle size of CLA-PTX NPs was significantly reduced when the CLA-PTX mixed DSPE-PEG DMSO solution (the ratio of CLA-PTX:DSPE-PEG = 1:0.1 (w/w)) was dropped in distilled water (Fig. 3A). In addition, when DSPE-PEG was used (the ratio of CLA-PTX:DSPE-PEG = 1:0.1 (w/w)), the CLA-PTX could still self-assemble forming CLA-PTX NPs when the concentration of CLA-PTX was as high as 30 mg/ml.

Also, the particle size of CLA-PTX NPs increased with the concentration and added volume of CLA-PTX DMSO solution (Fig. 3A).

### Effect of the added amount of DSPE-PEG on CLA-PTX NPs

It is well known that DSPE-PEG could be used as a carrier material to encapsulate the anti-cancer drugs. In order to confirm whether DSPE-PEG encapsulated CLA-PTX in the prepared CLA-PTX NPs, we investigated the effect of the added amount of DSPE-PEG on the particle size of CLA-PTX NPs. As shown in Fig. 3B and Table 1, when only a very low amount of DSPE-PEG was added to the CLA-PTX DMSO solution (CLA-PTX:DSPE-PEG = 1:0.0125, w/w), the particle size of the self-assembled CLA-PTX NPs was much smaller than that of DSPE-PEG-free CLA-PTX NPs. Also, the particle size of CLA-PTX NPs remained the same along with the ratio of CLA-PTX:DSPE-PEG from 1:0.0125 to 1:7 (w/w).

In contrast, we selected PTX as a control to investigate the effect of DSPE-PEG on PTX nanoparticle formation. The results indicated that PTX could not form nanoparticles at a lower concentration of DSPE-PEG (PTX:DSPE-PEG from 1:0.0125 to 1:20, w/w). At a high concentration of DSPE-PEG (PTX:DSPE-PEG, 1:40 or 1:80, w/w), the particle size of PTX micelles were observed at 241 or 118 nm (Fig. 3B and Table 1). In fact, PTX-loaded DSPE-PEG micelles had been investigated in our previous research involving preparation by a thin-film hydration method with PTX and DSPE-PEG at a ratio of 1:50 (w/w)^25^. It has been reported that many anti-cancer drug loaded DSPE-PEG micelles have been prepared by the thin-film hydration method with a high ratio of DSPE-PEG (drug:DSPE-PEG usually up to 1:10, w/w) and low drug loading efficiency (usually less than 10%)^26^,^27^. In the present research, we suggested that PTX-loaded DSPE-PEG micelles were formed at a high ratio DSPE-PEG (PTX:DSPE-PEG, 1:40 or 1:80, w/w). Also, the CLA-PTX-loaded DSPE-PEG micelles would also be formed at a high ratio DSPE-PEG (CLA-PTX:DSPE-PEG, 1:40 or 1:80, w/w).

Some pure hydrophobic drug nanoparticles are commonly stabilized by a small fraction of weakly binding surfactants, synthetic polymers, or biologically based macromolecules, such as a block copolymer poly(styrene)-b-poly(ethylene oxide) (PS-b-PEO) block copolymer stabilizing the β-carotene nanoparticles^28^,^29^.

Therefore, we suggested that DSPE-PEG could not encapsulate the CLA-PTX at the lower ration DSPE-PEG (CLA-PTX:DSPE-PEG from 1:0.0125 to 1:7, (w/w)). The role of DSPE-PEG is to prevent the CLA-PTX drop increasing or agglomerating when CLA-PTX self-assembled forming nanoparticles.

### Preparation of CLA-PTX@PEG NPs

**Figure 4:**
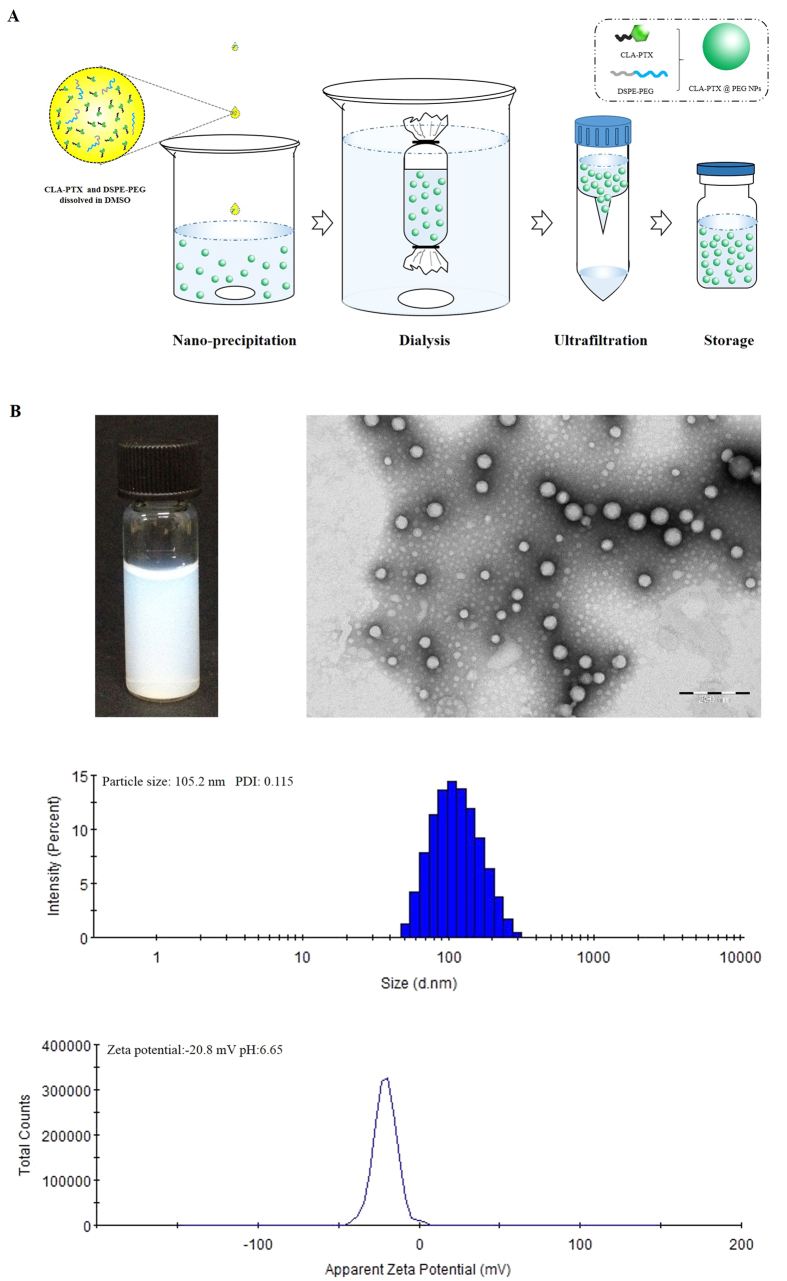
Schematic representation of CLA-PTX@PEG NPs preparation and characteristics of CLA-PTX@PEG NPs. (**A**) The schematic representation of CLA-PTX@PEG NPs preparation. (**B**) The appearance, TEM, particle size and zeta potential of CLA-PTX@PEG NPs.

**Figure 5:**
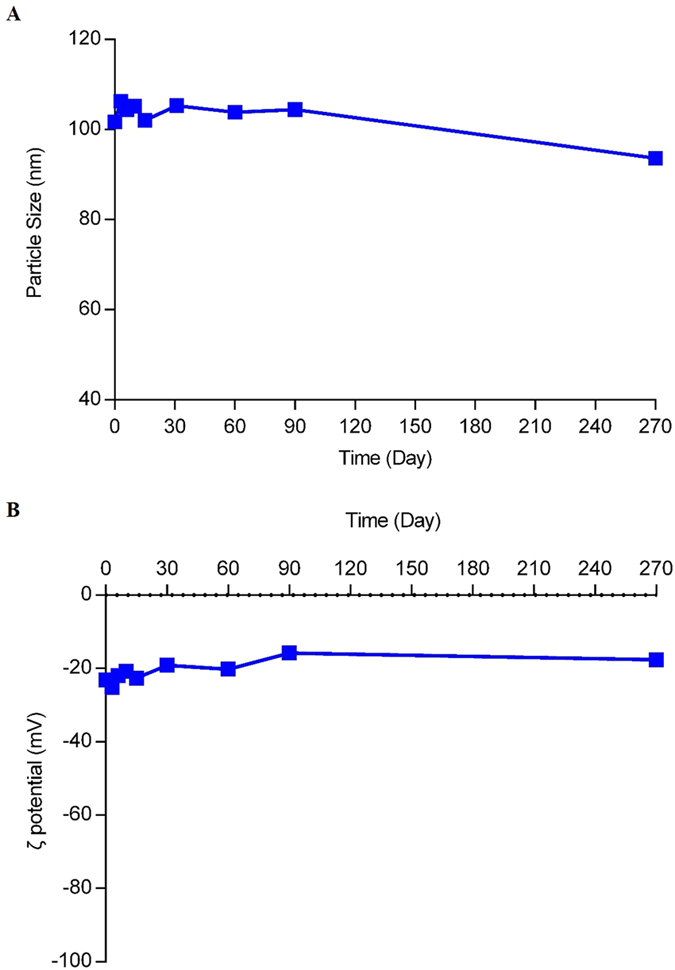
The particle size (**A**) and zeta potential (**B**) of CLA-PTX@PEG NPs within 9 months storage.

Based on CLA-PTX self-assembly forming nanoparticles and the role of DSPE-PEG, we prepared the CLA-PTX nanoparticles containing DSPE-PEG (CLA-PTX@PEG NPs) with 6 mg/ml of CLA-PTX. The drug loading of CLA-PTX in these nanoparticles was about 90% (w/w) with CrEL-free and organic solvent-free characteristics. Being the ratio of CLA-PTX:DSPE-PEG was only 1:0.1 (w/w), we suggested the prepared CLA-PTX@PEG NPs possessed carrier-free or drug-self carrier characteristic. The CLA-PTX@PEG NPs was prepared by using a simple precipitation method, as shown in Fig. 4A. The characteristics of CLA-PTX@PEG NPs are shown in Fig. 4B. The particle size of CLA-PTX@PEG NPs was 104.5 ± 2.2 nm (n = 3). CLA-PTX@PEG NPs exhibited a homogeneous distribution and good dispersibility in aqueous solution, confirmed by TEM images. The zeta potential of CLA-PTX@PEG NPs was −20 ± 2.77 mV. The pH value of CLA-PTX@PEG NPs was 6.65. In addition, the physical appearance, particle size and zeta potential of CLA-PTX@PEG NPs exhibited no significant changes after more than 9 months storage at room temperature, indicating the excellent stability of CLA-PTX@PEG NPs, as shown in Fig. 5.

It is well known that the concentration of PTX in commercial Taxol is 6 mg/ml and, considering the amount of excipients (CrEL and ethanol), the drug loading of PTX in Taxol is very low (about 1% w/w). In another PTX commercial formulation, Abraxane, a nanoparticle formulation with a particle size about 130 nm, the concentration of PTX was 5 mg/ml (consisting of human serum albumin 900 mg and PTX 100 mg, diluted with 20 ml 0.9% saline or 5% glucose infusion before clinical use). The drug loading of PTX in Abraxane is about 10% (w/w). In contrast, the concentration of CLA-PTX in our CLA-PTX@PEG NPs is 6 mg/ml and the drug loading of CLA-PTX in our CLA-PTX@PEG NPs is as high as 90% with CrEL-free and organic solvent-free characteristics.

We previous prepared a concentrated CLA-PTX microemulsion which containing dehydrated alcohol, Lipoid E 80, CrEL and soybean oil^19^. The concentration of CLA-PTX, CrEL, and Lipoid E80 in the concentrated microemulsion was 80, 95, and 190 mg/ml, respectively. The calculated content of CLA-PTX in the concentrated microemulsion was about 8% (w/v). The droplet size of CLA-PTX microemulsion after dilution with 5% glucose solution was about 176 nm. Considering the potential side effects of CrEL, we suggested the CLA-PTX@PEG NPs is superior to the CLA-PTX microemulsion because the CLA-PTX@PEG NPs contains only 10% DSPE-PEG.

### X-ray diffraction

**Figure 6:**
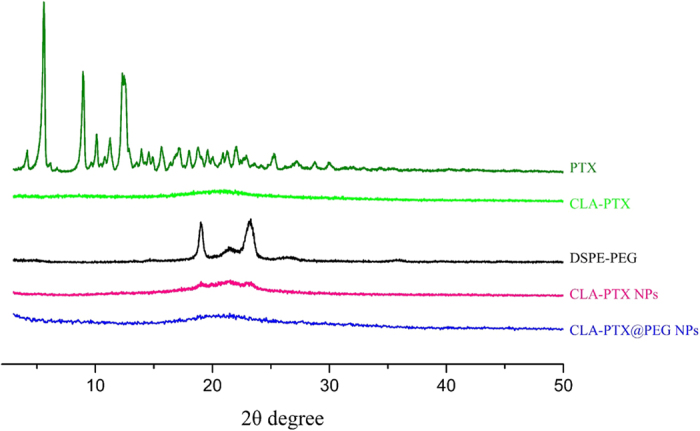
The X-ray diffraction spectra of PTX, CLA-PTX, DSPE-PEG, CLA-PTX NPs and CLA-PTX@PEG NPs.

The X-ray diffraction spectrum indicated that sharp and intense peaks of PTX were presented at a diffraction angle of 2θ 5.60, 8.96, 10.10, 11.24, 12.30, 15.64, 22.02°, suggesting that PTX was present in crystalline form. In contrast, there was no sharp peak attributable to the crystalline form of CLA-PTX and CLA-PTX NPs, suggesting that CLA-PTX, unlike PTX, was in an amorphous state, as shown in Fig. 6. Like CLA-PTX NPs, there was no sharp peak attributable to the crystalline form of CLA-PTX@PEG NPs, suggesting that CLA-PTX in CLA-PTX@PEG NPs was in an amorphous state, as shown in Fig. 6.

In addition, we determined the solubility of CLA-PTX in distilled water. Our results indicated that the solubility of CLA-PTX was less than 0.02 μg/ml, significantly lower than that of PTX (about 0.5 μg/ml). Considering that the HPLC retention time of CLA-PTX (about 22 min) was significantly longer than that of PTX (about 7.3 min), this suggests that the polarity of CLA-PTX is lower than that of PTX.

The PTX molecule allowed the growth of crystals in one direction, resulting in needle shaped crystals^30^. CLA-PTX is a conjugate with the 2′-OH of PTX connected to a carbon chain of CLA through an ester linkage (Fig. 1). The carbon chain in CLA-PTX reduces the solubility and polarity, compared with that of PTX, which prevents CLA-PTX crystal growth along the edge direction and promotes the self-assembly of nanoparticles^31^. Therefore, we suggested that the lower crystallinity, polarity and solubility of CLA-PTX compared with that of PTX might be the possible reason for CLA-PTX self-assembling forming nanoparticle. The DSPE-PEG used in CLA-PTX@PEG NPs could prevent the CLA-PTX drop increase or agglomerate when CLA-PTX self-assembled forming nanoparticles.

### Maximum tolerated dose (MTD) of CLA-PTX@PEG NPs in healthy mice

We investigated the MTD of CLA-PTX@PEG NPs in healthy ICR mice. The results indicated that the MTD of CLA-PTX@PEG NPs was up to 270 mg/kg, compared with 25–30 mg/kg for Taxol, indicating that the safety of CLA-PTX@PEG NPs was superior to that of Taxol. The blood cell levels of ICR mice after CLA-PTX@PEG NPs administration were also measured. As shown in Table 2, the level of WBC in the Taxol-treated group was significantly reduced compared with that in the 5% glucose group (*p* < *0.01*). In contrast, the level of WBC in the CLA-PTX@PEG NPs-treated group was similar to that in the 5% glucose group, indicating the safety of CLA-PTX@PEG NPs. In addition, the level of red blood cells (RBC) and platelets (PLT) in Taxol or CLA-PTX@PEG NPs-treatment groups was no significant difference with that in 5% glucose group.

### *In vitro* cellular uptake of CLA-PTX@PEG NPs

**Figure 7:**
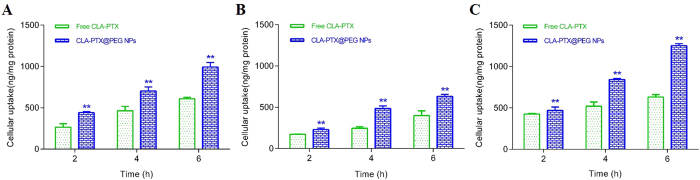
*In vitro* cellular uptake of the CLA-PTX@PEG NPs in MDA-MB-231 (**A**), B16-F10 (**B**) and U87-MG (**C**) cell lines. **p < *0.01*, vs Free CLA-PTX group. Free CLA-PTX: 40 mg CLA-PTX dissolved in 5 ml of CrEL/dehydrated ethanol (50:50, v/v), and then diluted with 5% glucose injection to 300 μM of CLA-PTX. This CLA-PTX solution was continue diluted with culture medium to 10 μM of CLA-PTX. CLA-PTX@PEG NPs: CLA-PTX@PEG NPs (6 mg/ml) diluted with 5% glucose injection to 300 μM of CLA-PTX. This CLA-PTX@PEG NPs was continue diluted with culture medium to 10 μM of CLA-PTX.

The *in vitro* cellular uptake of CLA-PTX@PEG NPs was examined in MDA-MB-231 (a human breast carcinoma cell line), B16-F10 (a murine melanoma cell line) and U87-MG (a human brain glioma cell line) cell lines. As shown in Fig. 7, CLA-PTX@PEG NPs exhibited enhanced cellular uptake in these three tumor cell lines compared with that of free CLA-PTX after an incubation of 2, 4, and 6 h, indicating that CLA-PTX@PEG NPs exhibited a higher uptake into tumor cells.

### *In vitro* cytotoxicity of CLA-PTX@PEG NPs

The *in vitro* cytotoxicity of CLA-PTX@PEG NPs was also evaluated in the MDA-MB-231, B16-F10 and U87-MG cell lines. The calculated IC_50_ values are displayed in Table 3. As shown in Table 3, the results indicated that the *in vitro* anti-tumor activity of CLA-PTX@PEG NPs in these three tumor cell lines was higher than that of free CLA-PTX (*p* < *0.05 or p* < *0.01*), but lower than Taxol (*p* < *0.01*). These results are consistent with those for the cellular uptake, suggesting that the higher cellular uptake of CLA-PTX@PEG NPs has the advantage of a greater *in vitro* anti-tumor effect than that of free CLA-PTX.

## Conclusion

In summary, we developed a novel self-assembling nanomedicine, CLA-PTX@PEG NPs, with higher drug loading and CrEL-free as well as organic solvent-free characteristics. The CLA-PTX@PEG NPs was prepared using a simple precipitation method. Being the ratio of CLA-PTX:DSPE-PEG was only 1:0.1 (w/w), we suggested the prepared CLA-PTX@PEG NPs possessed carrier-free characteristic. The particle size of CLA-PTX@PEG NPs was about 105 nm and the drug loading was about 90%. The stability results indicated that CLA-PTX@PEG NPs could be stored for at least 9 months. The safety of CLA-PTX@PEG NPs was demonstrated by the MTD results being CLA-PTX@PEG NPs with CrEL-free and organic solvents-free characteristics. CLA-PTX@PEG NPs exhibited a higher uptake into MDA-MB-231, B16-F10 and U87-MG tumor cells compared with that of free CLA-PTX. The anti-tumor activity of CLA-PTX@PEG NPs was higher than that of free CLA-PTX also confirmed by the *in vitro* experiments. The lower crystallinity, polarity and solubility of CLA-PTX compared with that of PTX might be the possible reason for CLA-PTX self-assembling forming nanoparticles, indicating a relationship between PTX modification and nanoparticle self-assembly. Overall, the data shown in this study strongly suggest that this drug self-delivery strategy based on self-assembly of a CLA-PTX conjugate offers a new way to prepare nanomedicine products for use in cancer therapy emphasizing the relationship between anticancer drug modification and self-assembly into nanoparticles.

## Additional Information

**How to cite this article**: Zhong, T. *et al*. A self-assembling nanomedicine of conjugated linoleic acid-paclitaxel conjugate (CLA-PTX) with higher drug loading and carrier-free characteristic. *Sci. Rep.* **6**, 36614; doi: 10.1038/srep36614 (2016).

**Publisher’s note:** Springer Nature remains neutral with regard to jurisdictional claims in published maps and institutional affiliations.

## Figures and Tables

**Table 1:** The effect of the added amount of DSPE-PEG on CLA-PTX NPs particle size.

	Ratio of weight (w/w)	Z-average Size (nm)	PDI	Size of Peak 1 (nm)	Size of Peak 2 (nm)	Size of Peak 3 (nm)
PTX:DSPE-PEG	1:80	118.1	0.198	2.597 (63.0%)	23.11 (22.2%)	161.6 (14.9%)
	1:40	241.2	0.374	2.279 (52.6%)	382.6 (23.8%)	24.11 (23.7%)
CLA-PTX:DSPE-PEG	1:80	102.40	0.216	2.664 (60.6%)	26.50 (20.3%)	179.8 (19.1%)
	1:40	180.10	0.283	2.304 (45.7%)	172.9 (31.9%)	20.79 (22.4%)
	1:20	142.20	0.236	230.3 (36.4%)	2.393 (32.6%)	24.28 (31.0%)
	1:10	441.40	0.501	101.4 (56.3%)	2.013 (24.0%)	16.32 (19.6%)
	1:7	82.23	0.163	96.44 (100.0%)		
	1:3	90.96	0.106	101.3 (100.0%)		
	1:2	84.71	0.175	102.8 (100.0%)		
	1:1	80.20	0.184	96.80 (100.0%)		
	1:0.33	77.78	0.149	92.71 (100.0%)		
	1:0.2	88.71	0.114	101.1 (100.0%)		
	1:0.15	82.67	0.095	92.62 (100.0%)		
	1:0.1	84.07	0.120	93.59 (100.0%)		
	1:0.05	76.24	0.100	84.20 (100.0%)		
	1:0.025	74.38	0.120	86.01 (100.0%)		
	1:0.0125	72.11	0.131	84.04 (100.0%)		
CLA-PTX NPs		105.9	0.090	117.3 (100.0%)		

**Table 2:** Blood cell levels in ICR mice after treatment with CLA-PTX@PEG NPs (n = 10).

Groups	WBC × 10^9^ /L^**a**^	RBC × 10^9^ /L^**a**^	PLT × 10^9^ /L^**a**^
5% glucose injection	4.63 ± 0.58	6.88 ± 0.67	504 ± 143
Taxol 25 mg/kg	2.60 ± 0.51^**^	8.27 ± 1.98	412 ± 67
CLA-PTX@PEG NPs 270 mg/kg	5.67 ± 1.01	7.40 ± 0.47	491 ± 81

**p < 0.01, vs 5% glucose injection group or CLA-PTX@PEG NPs 270 mg/kg group. ^a^WBC; white blood cells. RBC: red blood cells. PLT: platelets.

**Table 3:** The IC_50_ values of CLA-PTX@PEG NPs in in MDA-MB-231, B16-F10 and U87-MG cell lines. (n = 3).

Formulations	IC_50_ (μM)		
	MDA-MB-231 cells	B16-F10 cells	U87-MG cells
Taxol	1.51 ± 0.20	1.36 ± 0.17	2.85 ± 0.60
Free CLA-PTX	4.06 ± 0.21^$$^	4.14 ± 0.17^$$^	6.83 ± 0.16^$$^
CLA-PTX@PEG NPs	2.41 ± 0.52**^$$^	3.66 ± 0.40*^$$^	4.62 ± 0.59**^$$^

*p < 0.05 or **p < 0.01, vs Free CLA-PTX group; ^$$^*p* < 0.01, vs Taxol group.

Taxol: Taxol (6 mg/ml) was diluted with 5% glucose injection to 320 μM of PTX. This PTX solution was then diluted with culture medium to a series concentrations of PTX.

Free CLA-PTX: 40 mg CLA-PTX was dissolved in 5 ml of CrEL/dehydrated ethanol (50:50, v/v), and then diluted with 5% glucose injection to 320 μM of CLA-PTX. Following that, this CLA-PTX solution was diluted with culture medium to a series concentrations of CLA-PTX.

CLA-PTX@PEG NPs: CLA-PTX@PEG NPs (6 mg/ml) was firstly diluted with 5% glucose injection to 320 μM of CLA-PTX. This CLA-PTX@PEG NPs was then diluted with culture medium to a series concentrations of CLA-PTX.
